# Environmental and Population Biomonitoring of Selenium in Eastern Croatia

**DOI:** 10.3390/jox16040123

**Published:** 2026-07-02

**Authors:** Zvonimir Užarević, Martina Šrajer Gajdošik, Elvira Kovač-Andrić, Lidija Kalinić, Mihaela Vranješ Delać, Dinko Puntarić, Eda Puntarić, Domagoj Vidosavljević, Mario Begović, Vlatka Gvozdić

**Affiliations:** 1 Faculty of Education, University of Osijek, Cara Hadrijana 10, 31000 Osijek, Croatia; zuzarevic@foozos.hr; 2 Department of Chemistry, University of Osijek, Cara Hadrijana 8A, 31000 Osijek, Croatia; msgajdosik@kemija.unios.hr (M.Š.G.); eakovac@kemija.unios.hr (E.K.-A.); 3 Department of Biology, University of Osijek, Cara Hadrijana 8A, 31000 Osijek, Croatia; lkalinic@biologija.unios.hr; 4 Faculty of Veterinary Medicine, University of Zagreb, Heinzelova 55, 10000 Zagreb, Croatia; mihaela.vranjes@hotmail.com; 5 Public Health Institute of Karlovac County, Dr. Vlatka Mačeka 48, 47000 Karlovac, Croatia; dinko.puntaric2@gmail.com; 6 Ministry of Environmental Protection and Green Transition of the Republic Croatia, Radnička Cesta 80, 10000 Zagreb, Croatia; edapuntaric@gmail.com; 7 Faculty of Medicine, University of Osijek, Josipa Huttlera 4, 31000 Osijek, Croatia; domagoj.vidosavljevic@gmail.com; 8 Faculty of Medicine, Pharmacy and Health, European University Brčko District, Bijeljinska Cesta 72–74, 76100 Brčko, Bosnia and Herzegovina; begovicvu@gmail.com

**Keywords:** selenium, biological samples, soil, dandelion, eastern Croatia

## Abstract

Selenium is a trace element of vital importance for ecosystem functioning and human health mainly due to its antioxidant and protective properties. Since both selenium deficiency and excess can have harmful effects on living organisms, monitoring its distribution in biological systems and the environment is of significant scientific and public health interest. This study systematically assessed selenium concentrations in biological (urine, serum, and hair) and environmental (soil and dandelion) samples in eastern Croatia. Selenium concentrations were determined using the inductively coupled plasma mass spectrometry method. In biological samples, median selenium concentrations were from 15.02 to 37.15 μg·L^−1^ in urine, from 80.76 to 114.05 μg·L^−1^ in serum, and from 0.21 to 0.46 µg·g^−1^ in hair. In environmental samples, median selenium concentrations varied depending on location, ranging from 0.32 to 0.51 mg·kg^−1^ in soil and from 6.55 to 84.41 µg·kg^−1^ in dandelion. PCA was applied to identify overall patterns and groupings among biological and environmental samples, showing that dandelion samples from urban locations exhibited the highest selenium concentrations, indicating the influences of urban environmental conditions on selenium accumulation.

## 1. Introduction

Selenium is a trace element of great importance for the human body due to its vital role in various biological processes including oxidative stress response and immune regulation [1]. Selenium is essential for the synthesis of selenocysteine, an uncommon amino acid which is an important constituent of selenoproteins. These proteins are involved in key physiological processes such as the regulation of the immune system, antioxidant defense and thyroid hormone metabolism [1,2,3]. Glutathione peroxidases, thioredoxin reductases and iodothyronine deiodinases are among the 25 human selenoproteins that have been identified to date. Since the main functions of these proteins are connected to the maintenance of redox equilibrium and protection against oxidative damage in cells, the importance of sufficient selenium intake for maintaining optimal cell function and overall health is increasingly recognized [2,4]. Studies have shown that adequate selenium levels may reduce the risk of various diseases, including certain cancers, cardiovascular diseases, viral infections, male infertility and affective disorders [5,6,7,8]. However, it is important to maintain appropriate selenium levels because excessive levels can lead to toxicity, which can manifest as gastrointestinal distress and a characteristic garlic-like odor [9]. Recent findings have also shown that both selenium deficiency and excess levels can contribute to the pathogenesis of metabolic conditions such as type 2 diabetes mellitus and metabolic dysfunction-associated steatotic liver disease (MASLD) [10,11]. Accordingly, the recommended daily intake for selenium for healthy adults has been set at 55–70 µg·day^−1^ [12].

Selenium can enter the environment from natural sources, such as the weathering of rocks and volcanic eruptions, but also from numerous human activities. The main anthropogenic sources of selenium in the environment are mining, metallurgy, coal combustion, oil refining, industrial production and various agricultural processes. Elevated concentrations of selenium can lead to soil, water and air pollution and pose a serious risk to human health and ecosystems. Therefore, controlling emissions, as well as the appropriate regulation of industrial and agricultural processes, is essential in order to prevent the harmful effects of selenium in the environment [13,14,15]. The distribution of selenium in soil and food varies greatly and depends mainly on the characteristics of the soil itself, i.e., the content of organic matter, pH and redox status. In the diet, selenium mainly comes from protein-rich foods such as meat, fish and eggs, while cereals, fruits and vegetables usually contain lower concentrations of this element [5]. One of the key aspects that determine the nutritional value, but also the toxicity of selenium, is the form (chemical speciation) in which it occurs in nature. Organic selenium compounds such as selenomethionine and selenocysteine are generally more bioavailable and less toxic than inorganic forms (selenite and selenate) [14,15,16]. Despite increasing awareness of healthy eating and emphasis on the importance of supplementation, insufficient selenium intake is still present in many countries across Europe, including Belgium, Poland, Slovenia, and Turkey [17].

Previous studies in eastern Croatia have reported selenium deficiency in livestock meat, soils, and cereals [18,19]. Comparative analyses of foodstuffs from two regions of eastern Croatia, northern (Drava River basin: Ivanovci and Zelčin) and southern (Sava River basin: Berbina and Slavonski Kobaš), revealed substantial regional variability, with protein-rich foods showing the highest selenium levels and fruits and vegetables the lowest [18]. These concentrations were comparable to those reported in other European countries, with slightly higher selenium levels observed in foods produced near the Sava River. Limited studies in human populations have shown mean serum selenium concentrations of approximately 66.8 ± 14.4 µg·L^−1^ in healthy adults from the Zagreb area, whereas significantly lower concentrations were reported in patients with colorectal adenoma and carcinoma.

In humans, serum, hair and urine are considered reliable indicators of selenium status in the body. In urine, the amount of selenium primarily reflects its short-term intake, as it is dependable on the daily diet and fluid intake. In contrast, the levels of selenium in serum indicate medium- to long-term exposure. Hair and nails are the least susceptible to short-term changes of selenium exposure. Therefore, selenium concentrations in hair and nails are considered the most reliable indicators of a long-term intake. However, selenium content in hair can be influenced by various environmental factors, dietary habits and the use of cosmetic products containing selenium [20,21]. Recent advances in analytical techniques, particularly the use of inductively coupled plasma mass spectrometry (ICP-MS) and its combination with high-performance liquid chromatography (HPLC-ICP-MS), now allow for the determination of the total amount and chemical forms of selenium in biological and environmental samples with great precision, even at very low concentrations [22].

No systematic research has been conducted to date that would comprehensively encompass the assessment of selenium concentrations in biological samples (serum, urine and hair) together with environmental matrices (soil, water and plants) in the area of eastern Croatia. The analysis of soil, plant, and biological samples was intended to provide a comprehensive overview of selenium distribution across environmental and human compartments. To our knowledge, no previous study in the investigated area has simultaneously combined these matrices within a single integrated approach. Dandelion (*Taraxacum officinale* L.) was selected as a representative wild-growing species that is not typically subjected to agricultural fertilization practices, thereby providing a more realistic indication of environmental contamination. This integrated design was aimed at enabling a more complete understanding of selenium occurrence and its potential transfer from soil through plants to humans.

Therefore, the aim of this work is to determine selenium levels in the aforementioned samples in order to assess the selenium status and possible exposure pathways of the population of eastern Croatia.

## 2. Materials and Methods

### 2.1. Sample Collection and Analysis

**Figure 1:**
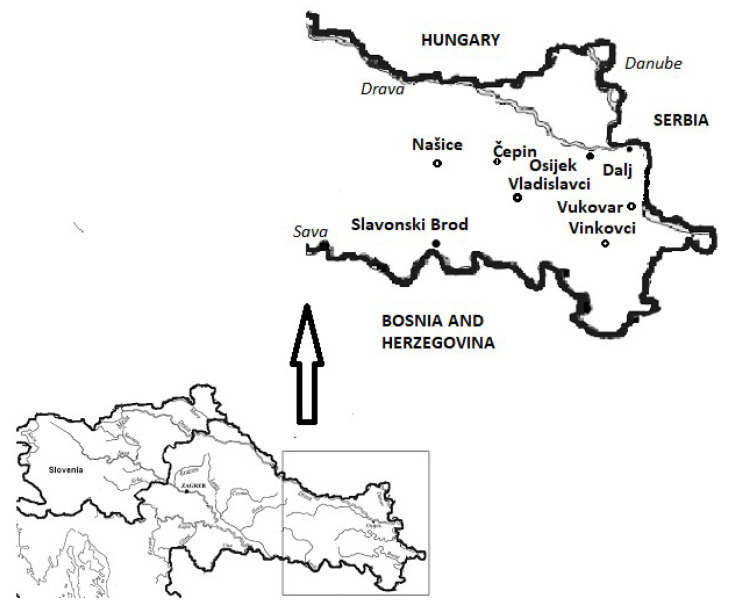
Map of investigated locations.

Sample collection was conducted in eight settlements across eastern Croatia, comprising five cities (Vukovar, Vinkovci, Slavonski Brod, Našice and Osijek) and three villages (Dalj, Vladislavci and Čepin) (Figure 1). Biological samples (urine, serum, and hair) were collected from a total of 500 participants. At these locations, environmental samples, namely, soil and dandelion (*Taraxacum officinale* L.), were also collected.

Selenium concentrations in biological and environmental samples were determined at the Analytical Toxicology and Mineral Metabolism Unit, Institute for Medical Research and Occupational Health, Zagreb, Croatia, using an Agilent 7500 cx inductively coupled plasma mass spectrometer (ICP-MS) (Agilent Technologies, Tokyo, Japan). The ICP-MS instrument was equipped with an I-AS autosampler, a MicroMist nebulizer, a Scott spray chamber, Ni cones and an octopole reaction cell. For Se determination, ^78^Se and ^74^Ge as internal standard (2 µg·L^−1^) were monitored. Hydrogen (purity 99.99 vol%) was used as the reaction gas at a flow rate of 3.6 ml·min^−1^.

Serum samples were quantified using matrix-matched calibration. Calibration standards for urine and digested hair, dandelion, and soil samples were prepared in 1% HNO_3_. PlasmaCAL Se single-element calibration solution (1 g·L^−1^; SCP Science, Montreal, QC, Canada) was used. Instrument sensitivity was optimized using a tuning solution containing 1 g·L^−1^ of Li, Co, Y, Ce and Tl. Prior to analysis, serum and urine samples were diluted 1:20. Serum samples were diluted with a solution containing 0.7 mM NH_3_, 0.01 mM EDTA, and 0.07% (*v*/*v*) Triton X-100 (TX-100), whereas urine samples were diluted with 1% HNO_3_.

Hair, dandelion, and soil samples were digested using an UltraClave IV high-pressure microwave digestion system (Milestone, Bergamo, Italy). HNO_3_ (65%, p.a, Merck, Darmstadt, Germany) for digestion was purified using a duoPUR sub-boiling distillation system (Milestone, Bergamo, Italy).

After washing with acetone, hair samples were digested with 4 mL of purified concentrated HNO_3_ and ultrapure water (1:1, *v*/*v*). Following digestion, samples were brought to a final mass of 6 g with ultrapure water (GenPure, TKA System Gmbh, Niederelbert, Germany), stored at +4 °C, and further diluted 40-fold prior to analysis. Lyophilized dandelion samples (0.15 g) were digested with 4 mL purified concentrated HNO_3_ and ultrapure water (1:1, *v*/*v*), adjusted to 6 g with ultrapure water, and diluted 5-fold with 1% HNO_3_ before analysis. Soil samples (0.1 g) were digested with 4 mL of purified concentrated HNO_3_ and ultrapure water (1:1, *v*/*v*), with the addition of 0.2 mL of 47% HF. After digestion, samples were adjusted to 10 g with ultrapure water and further diluted 15-fold with 1% HNO_3_ prior to ICP-MS analysis.

Measurement accuracy was verified using certified reference materials and quality control materials, including ClinChek Serum Control for Trace elements (Levels I and II; RECIPE Chemicals + Instruments, GmbH, Munich, Germany), Seronorm Trace Element Serum (Levels I and II), Urine (Sero, Billingstad, Norway), IAEA-086 Human Hair and IAEA-SL-1 Lake Sediment (IAEA, Vienna, Austria), NIST SRM 1577a Bovine Liver, NIST SRM 2709 San Joaquin Soil, and NIST SRM 1570a Spinach Leaves (NIST, Gaithersburg, MD, USA). The limits of detection (LODs) and limits of quantification (LOQs) were: serum (1.5 µg·L^−1^; 3.8 µg·L^−1^), urine (1.5 µg·L^−1^; 2.6 µg·L^−1^), hair (0.02 µg·g^−1^; 0.03 µg·g^−1^), soil (0.07 mg·kg^−1^; 0.1 mg·kg^−1^), and dandelion (0.008 µg·kg^−1^; 0.012 µg·kg^−1^).

### 2.2. Statistical Data Analysis

Statistical data analysis was performed using descriptive statistical methods. The normality of data distribution was assessed using the Shapiro–Wilk test. Nonparametric tests (Kruskal–Wallis tests) were applied for comparison between groups (*p* = 0.05). In addition to univariate analyses, a multivariate statistical approach was applied to explore potential relationships among the analyzed variables. The principal component analysis (PCA) method was used to identify correlations between variables (selenium concentrations in biological samples) and objects (sampling locations), as well as to provide a simplified visual representation of the relationships observed in the dataset. All statistical analyses were conducted using the software package Statistica 14.0 (TIBCO, Palo Alto, CA, USA)**.** A significance level of *p* < 0.05 was considered statistically significant.

## 3. Results

### 3.1. Descriptive Statistics

Table 1, Table 2 and Table 3 present the results of basic statistical analysis, including the median, 25th and 75th percentiles, minimum and maximum concentrations measured in urine, serum and hair samples. Table 4 and Table 5 summarize the selenium concentrations determined in environmental samples, soil and dandelion.

### 3.2. Kruskal–Wallis Test

**Figure 2:**
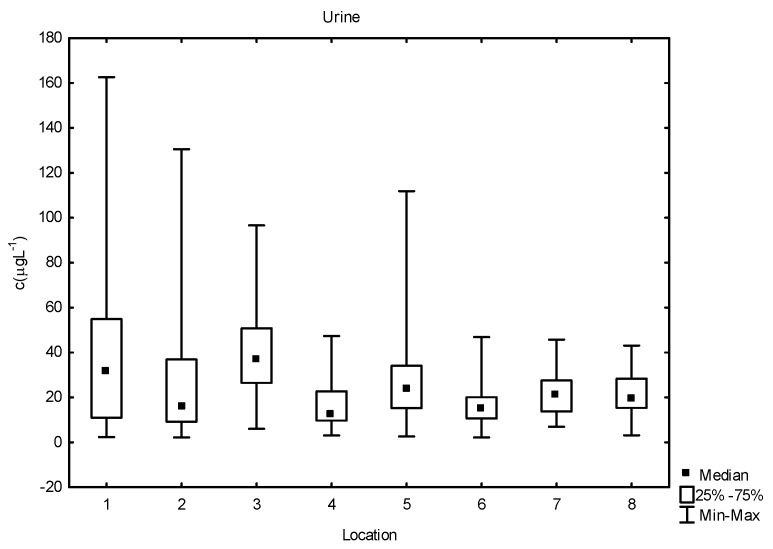
Kruskal–Wallis analysis of selenium concentrations in urine samples across sampling locations (1—Vladislavci; 2—Dalj; 3—Čepin; 4—Našice; 5—Osijek; 6—Vukovar; 7—Slavonski Brod; 8—Vinkovci).

**Figure 3:**
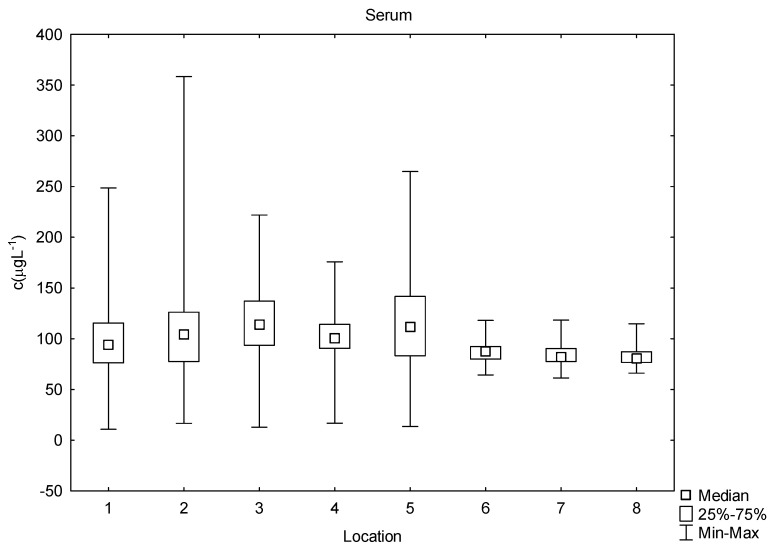
Kruskal–Wallis analysis of selenium concentrations in serum samples across sampling locations (1—Vladislavci; 2—Dalj; 3—Čepin; 4—Našice; 5—Osijek; 6—Vukovar; 7—Slavonski Brod; 8—Vinkovci).

**Figure 4:**
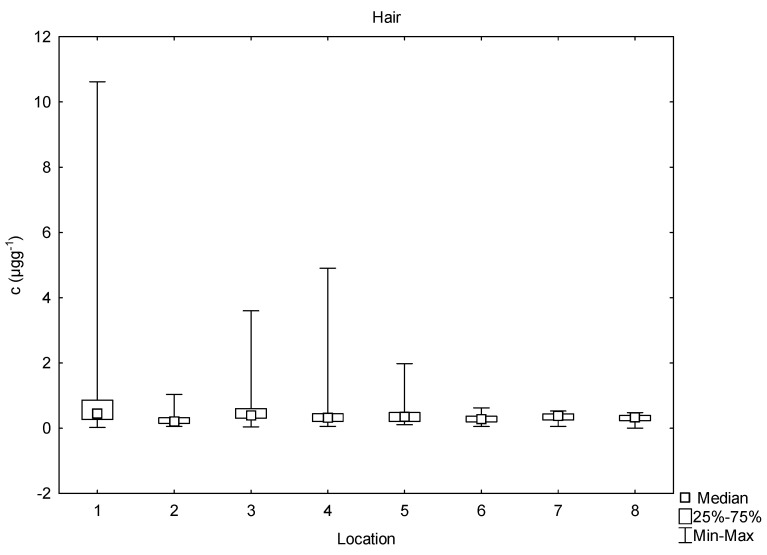
Kruskal–Wallis analysis of selenium concentrations in hair samples across sampling locations (1—Vladislavci; 2—Dalj; 3—Čepin; 4—Našice; 5—Osijek; 6—Vukovar; 7—Slavonski Brod; 8—Vinkovci).

The results of the nonparametric Kruskal–Wallis test are presented in Figure 2, Figure 3 and Figure 4 while the results of post-hoc analyses are included in the Supplementary Materials (Tables S1–S3). For the urine samples, the highest number of statistically significant differences was observed between location Čepin and most of the remaining sites (Figure 2). The *p* values ranged from 0.00 in Dalj, Našice, and Vukovar to 0.021, 0.027 and 0.039 in Vinkovci, Slavonski Brod and Osijek (*p* = 0.05).

In contrast to urine, serum exhibits a completely different pattern. The highest number of statistically significant differences was observed between the three cities (Vinkovci, Vukovar and Slavonski Brod) and the remaining locations (Figure 3). The *p* values ranged from 0.00 to 0.04 (*p* = 0.05).

With the exception of Vladislavci, Vukovar and Vinkovci, the highest number of statistically significant differences for hair samples were observed between the Dalj location and the remaining sites (Figure 4). The *p* values ranged from 0.001 to 0.006 (*p* = 0.05).

### 3.3. Cluster Analysis

**Figure 5:**
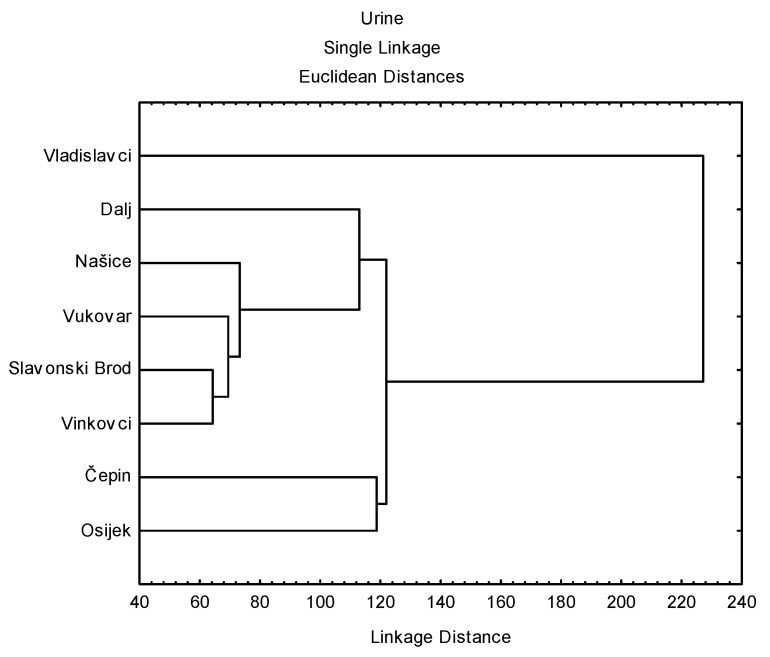
The results of cluster analysis (urine).

**Figure 6:**
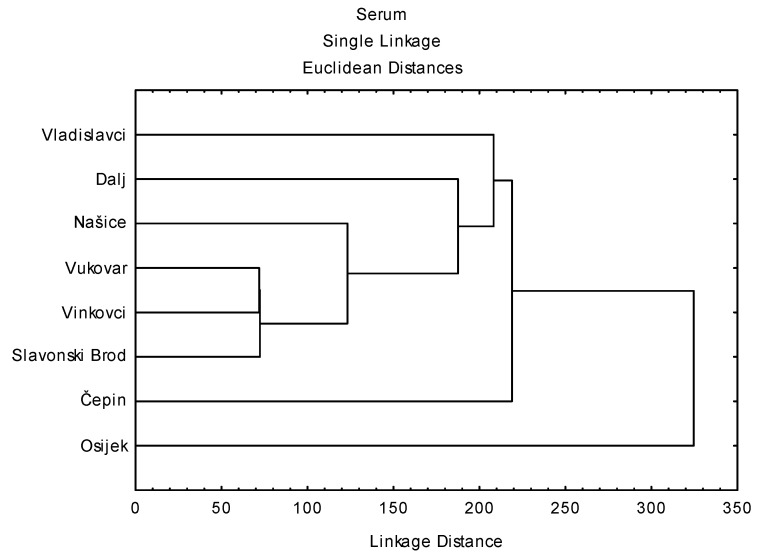
The results of cluster analysis (serum).

**Figure 7:**
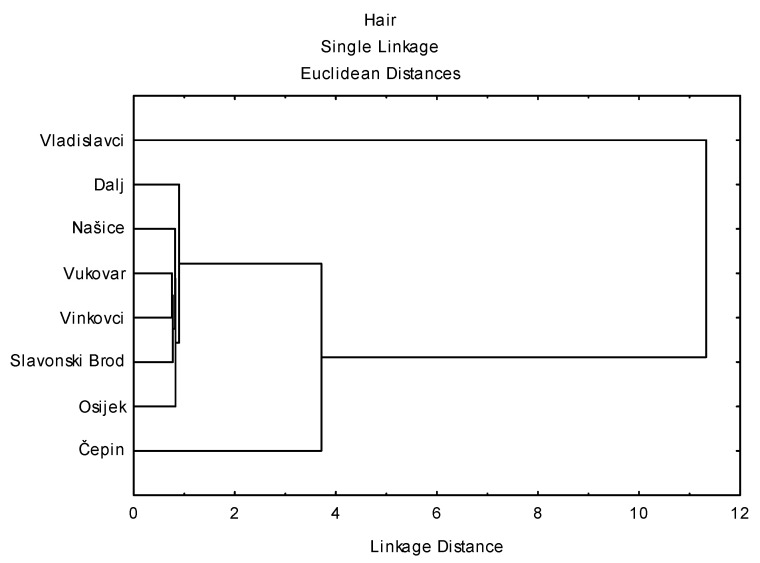
The results of cluster analysis (hair).

Since the results of the descriptive statistics provide only a one-dimensional view of the data, a multivariate method and cluster analysis was applied. Figure 5, Figure 6 and Figure 7 present the results of the cluster analysis, displayed in the form of dendrograms. The dendrogram obtained from the cluster analysis of selenium concentrations in urine shows the presence of two clusters: a single-member cluster consisting of Vladislavci, characterized by the highest 75% percentile values, and a complex cluster comprising all remaining locations. Urban locations (Vukovar, Vinkovci, Slavonski Brod, Našice and Osijek) predominantly formed a separate cluster, although some overlap with other sites was observed (Figure 5).

The dendrogram resulting from the cluster analysis of selenium concentrations in serum revealed the presence of a complex cluster comprising most study sites and a distinct single-member cluster represented by Dalj (Figure 6).

The results of the cluster analysis based on selenium concentrations in hair are presented in Figure 7. The analysis revealed a complex cluster comprising Dalj, Vukovar, Slavonski Brod, Vinkovci, Osijek and Našice. In contrast, Vladislavci and Čepin formed separate, single-member clusters. The distinction of these two locations from the remaining study sites can be attributed to their higher median and 75% percentile values compared with the other locations. The results of the cluster analysis indicated that rural locations were mostly separated from urban sites.

### 3.4. Principal Component Analysis

**Figure 8:**
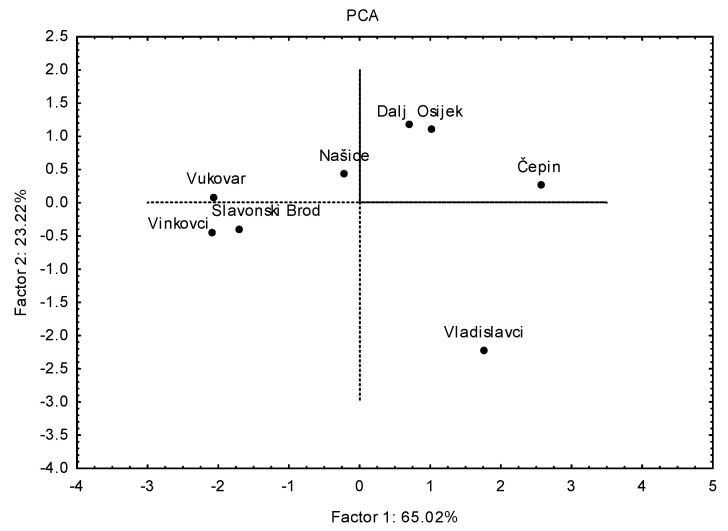
The results of PCA (locations).

**Figure 9:**
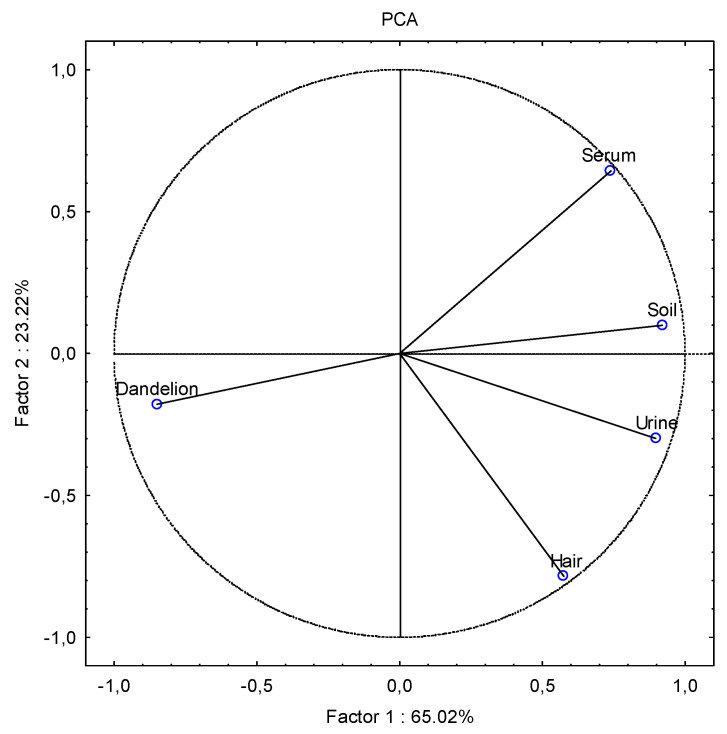
The results of PCA (variables).

PCA and cluster analysis provide different, yet complementary, insights into the data. While cluster analysis groups samples or variables based on similarity, PCA enables dimensionality reduction and the identification of the main sources of variability within the dataset. PCA not only reveals which variables contribute most to the differences between samples but also allows the visualization of continuous patterns and trends, which cluster analysis alone does not provide. In this way, PCA can assist in interpreting the data structure and in identifying key factors influencing clustering, thereby complementing the information obtained through cluster analysis. The first two components explained about 88% of the total variance, making them sufficient to represent the data in two component plots (Figure 8 and Figure 9).

The PCA results indicate the grouping of three cities (Vukovar, Vinkovci and Slavonski Brod) located in two distinct counties characterized by lower selenium concentrations in biological samples, but higher concentrations in plants, while the remaining locations around the city of Osijek were associated with higher selenium concentrations in biological samples and soil.

## 4. Discussion

Serum selenium is considered a reliable biomarker of selenium status because it reflects circulating selenoproteins, particularly selenoprotein P and glutathione peroxidase, and is correlated with functional antioxidant activity. Unlike urine, which primarily reflects recent dietary intake and can fluctuate widely with hydration and excretion rates, serum provides a more stable measure of selenium availability in the body. Similarly, while hair and nails can indicate long-term selenium accumulation, their selenium content is influenced by external contamination and variable growth rates, making them less precise for assessing current nutritional functional status. For these reasons, serum selenium remains the preferred biomarker in population studies and clinical assessments. In the adult Croatian population, serum selenium median values were from 80.75 to 114.05 µg·L^−1^. Studies conducted in 2004 indicated lower serum selenium concentrations, with an average value of 66.8 µg·L^−1^, which was below the average reported in other EU countries at the time [23,24]. However, selenium status in neighboring countries shows considerably variability, with some populations exhibiting lower (Serbia, 66.5 µg·L^−1^ [25]; Hungary, 77.4 µg·L^−1^ [26]; and Slovenia, 62–66 µg·L^−1^ [27]) or comparable selenium levels to those reported in our population (Bulgaria, 83.2 µg·L^−1^) [28]. In central Europe, serum concentrations in the Czech Republic were approximately 63 µg·L^−1^ [29]. The rest of the European population generally showed higher concentrations, with levels comparable to these observed in the present study: France, approximately 86–90 µg·L^−1^; Sweden, 75.6 µg·L^−1^ [30]; Italy, 140 µg·L^−1^ [31]; and Germany, approximately 132 µg·L^−1^ [32]. The serum selenium concentrations observed in this study are generally within the range considered adequate for European populations. Previous studies have suggested a practical reference value of approximately 80 µg·L^−1^ as the lower limit of optimal selenium status in adults [33,34].

In addition to serum measurements, selenium was determined in urine and hair to assess short-term and long-term selenium exposure, respectively, complementing the functional information provided by serum selenium. While urinary selenium is not directly associated with the activity of selenoenzymes, it remains a useful biomarker for monitoring recent selenium intake and regional differences in selenium status. In a Norwegian cross-sectional study of adults following vegan, lacto-ovo vegan or pescatarian diets, the median urinary selenium concentration was 13 µg·L^−1^ (interquartile range, 6–22 µg·L^−1^) reflecting the recent intake and excretion patterns of selenium in this population [35]. In our study, urinary selenium concentrations ranged from 15.02 to 37.15 µg·L^−1^, which is generally consistent with values reported in healthy European populations, such as 15–19 µg·L^−1^ in Germany, 15.2 µg·L^−1^ in the United Kingdom, 21.6 µg·L^−1^ in Belgium, and 14.1 µg·L^−1^ in Slovenia [36]. Among eight different locations, the highest selenium concentrations in both serum and urine were observed in one village (Čepin). Water samples from Čepin were analyzed for selenium content, with all values (max = 0.52 µg·L^−1^) below the maximum allowable concentration of 10 µg·L^−1^ as set by Croatian drinking water regulations. Considering the low selenium content in drinking water from Čepin, the observed selenium levels did not correlate with water selenium concentrations.

Selenium is an essential trace element involved in critical biological processes, including antioxidant defense, thyroid hormone metabolism and immune function. Human hair has been widely used as a biomarker of selenium status because it reflects cumulative intake over time. Since selenium is incorporated into hair during growth, hair concentrations provide insight into long-term nutritional status and environmental exposure. Several studies reported reference values of selenium in hair across different populations. In northern Poland, Hać et al. (2002) reported mean hair selenium concentrations of 0.30 µg·g^−1^ in healthy adults, with a significant positive correlation between hair and plasma selenium, supporting hair as a reliable biomarker of selenium status [21]. Lorenzo Alonso et al. (2005) reported a broader range of 0.22–1.50 µg·g^−1^ in mixed populations [37]. In Algeria, a study by NNA analysis (2021) on control subjects showed 0.39 µg·g^−1^ [38], while children in selenium-deficient regions of Tibet exhibited lower mean levels of approximately 0.23 µg·g^−1^ [39]. In Moldova, Kapitalchuk et al. (2023) found hair selenium concentrations ranging from 0.14 to 5 µg·g^−1^, highlighting intra- and interregional variability due to environmental and dietary factors [40]. Overall European populations typically show hair selenium concentrations between 0.3 and 0.6 µg·g^−1^, though deviations can occur depending on dietary intake and environmental selenium availability. An analysis of hair samples from 408 residents across 10 provinces in China showed that most selenium concentrations ranged from 0.30 to 0.45 µg·g^−1^. In a recent study conducted in Poland, trace element concentrations in scalp hair were analyzed in patients with preserved ventricular ejection fraction compared to a healthy control group. Special attention was given to selenium, with measured levels ranging from 0.37 to 0.42 µg·g^−1^ [41]. The results of our analysis showed that selenium concentrations ranged from 0.21 to 0.46 µg·g^−1^ with percentile values from 0.15 to 0.86, falling within the normal hair selenium range reported by Rodoushkin et al. [42,43]; these were obtained from areas unaffected by anthropogenic activities, reflecting natural background levels. The highest 75th percentile concentrations in hair (and urine) were again detected in a settlement (Vladislavci) close to that (Čepin) in which the highest median urine concentrations had previously been observed. The concentrations were within the background ranges reported in the literature for areas without anthropogenic influence and therefore do not indicate environmental contamination.

Due to its essential biological function and its role linking soil, plants, and humans, selenium is considered as a trace element of significant geochemical and nutritional importance. Soil selenium concentrations are highly variable and depend on parent geology, soil type, organic matter, content and anthropogenic influences. Surface soils exhibit selenium concentrations in the range of 0.01 to 2 mg·kg^−1^, with many European soils toward the lower end of this range. The global average soil selenium concentration is 0.32 mg·kg^−1^. Lower soil selenium can limit uptake by crops and consequently contribute to suboptimal selenium intake in humans and livestock, particularly where diets are heavily based on cereals or lack diversification [44]. Studies of European soils reveal considerable variability [45]. Recent studies from Southeast Europe have shown considerable spatial variability in soil selenium concentrations, with many soils exhibiting levels below deficiency thresholds. Only soils from the Mostar area (Bosnia and Herzegovina) had selenium concentrations above deficit levels (0.5 mg·kg^−1^) [46]. In Croatia, national and regional studies reflect similarity diverse soil selenium status. In the vicinity of Koprivnica in north-western Croatia, an investigation showed relatively low to moderate content, which may limit selenium transfer to crops [47]. In the Istrian Raša region, Medunić et al. (2021) documented significantly elevated soil selenium concentrations, up to 10 mg·kg^−1^, associated with local anthropogenic influences, demonstrating that localized contamination can greatly exceed typical background levels [48]. The relatively low soil selenium status in large parts of Croatia (e.g., Osijek, its vicinity and north-western region) has implications for crop selenium content and human and animal selenium nutrition [46]. These findings highlight the importance of considering soil geochemistry and local anthropogenic factors when assessing selenium availability and nutritional risk in Croatia and similar European settings. This study was conducted at eight different locations, namely, two urban centers (Osijek, 0.39 mg·kg^−1^; Našice, 0.38 mg·kg^−1^), three other city centers with lower values (Vukovar, 0.32 mg·kg^−1^; Vinkovci, 0.37 mg·kg^−1^; Slavonski Brod, 0.33 mg·kg^−1^) and three villages: Dalj (0.46 mg·kg^−1^), Vladislavci (0.42 mg·kg^−1^) and Čepin (0.51 mg·kg^−1^). Soil selenium concentrations were within a relatively small range of 0.32 to 0.51 mg·kg^−1^, with slightly lower values observed in urban centers and slightly higher values in the agricultural villages of Čepin, Dalj and Vladislavci. Lower selenium concentrations in cities are likely due to reduced organic matter content, mixed soil, decreased microbial activity and limited natural geochemical replenishment, whereas agricultural soils in villages provide more favorable geochemical conditions for selenium retention. The results indicate that soils in the studied area of eastern Croatia have low to moderate selenium availability, which may limit accumulation in crops and consequently affect human and animal nutrition [49]. Soils in eastern Croatia exhibit selenium concentrations ranging from 0.32 to 0.51 mg·kg^−1^, indicating a moderate to relatively well preserved selenium status compared to other areas in the region and Europe. The soils of Central Europe, mainly in Slovakia, are poor in selenium (less than 0.2 mg·kg^−1^) [50]. The plow layer soils in Sweden showed a median selenium concentration of 0.23 mg·kg^−1^, while analysis in Scottish arable topsoils showed that they contained between 0.19 and 1.46 mg·kg^−1^, with a mean of 0.63 mg·kg^−1^ [51]. Compared to northern Serbia (Vojvodina), where average concentrations are around 0.20–0.25 mg·kg^−1^, soils in eastern Croatia show higher selenium content. The range reported for Mostar and northern Bosnia and Herzegovina (0.32–0.68 mg·kg^−1^) locates eastern Croatia soils within the mid-range of the regional spectrum. In the context of the European average (0.35–0.45 mg·kg^−1^), soils in eastern Croatia are positioned in the upper part of the range.

*Taraxacum officinale* L., commonly known as dandelion, is a widely distributed herb that is available throughout the year, making it suitable for environmental analyses. Its leaves, roots and flowers are consumed as food, providing vitamins and minerals, and dandelion has antioxidants and probiotic compounds such as inulin. Medicinally, dandelion has been documented to exhibit diuretic, hepatoprotective, anti-inflammatory and antioxidant effects. In addition, it serves as a bioindicator for soil health and trace element bioavailability, including selenium [52]. In Croatia, selenium accumulation in plants has been documented in areas affected by unique geochemical conditions. Medunić et al. [48] investigated vegetables, fruits and wild plants in the Raša Bay area (Istrian Peninsula) where coal-derived selenium in water and soil is elevated. They reported that selenium levels in garden vegetables from this region were elevated up to 5- and 20-fold greater in comparison to typical Croatian (cabbage 8.0 µg·kg^−1^) or Greek (6.5 µg·kg^−1^) selenium levels in vegetables [18,48,53].

The selenium concentrations in dandelion sampled at five locations (Osijek, Našice, Vladislavci, Čepin and Dalj) are similar to those published by Kabata-Pendias [46]. However, the values measured in three cities (Vinkovci, Vukovar and Slavonski Brod) are several times higher. Pollution with selenium is commonly observed in industrial areas where it is released into the atmosphere as a result of metal processing activities and coal combustion. Additionally, fly ash generated from refuse incineration has been identified as a significant source of selenium. Also, industrial and agriculture production, coal combustion, oil refining, fossil fuel usage, pesticide manufacturing, mining and metallurgy are examples of anthropogenic sources [13]. There is a significant difference between the three cities (Vinkovci, Vukovar and Slavonski Brod), where elevated concentrations were found, and the remaining locations regarding the method of dandelion collection for analysis. While in the three cities samples were collected in the city center, close to traffic areas, at the remaining locations (Osijek, Našice, Čepin, Dalj and Vladislavci) they were collected in areas further away from the city or village centers. Elevated selenium concentrations in dandelion leaves cannot be directly correlated with increased selenium concentrations in the soil, as soil selenium levels show minimal variation across the investigation area of eastern Croatia. The main pedological difference within eastern Croatia is the predominance of soils originating from Chernozems developed from loess in the Osijek and Vukovar area, whereas hydromorphic and alluvial soils occur more frequently in the Slavonski Brod area, reflecting differences in geomorphological and hydrological conditions. In addition, previous studies conducted in eastern Croatia have demonstrated considerable spatial variability in soil properties, with soil samples ranging from slightly acidic to acidic and generally containing low to moderate amounts of organic matter. Both parameters are well known to influence selenium mobility and plant availability. Urban areas are exposed to a wide range of anthropogenic inputs, including urban environmental contamination, which may contribute to selenium deposition in the local environment. A variety of different compounds including selenium are added to vulcanize rubber and achieve a highly elastic material [54]. A study conducted in an urban traffic area in India found that tire wear contributed 31% to road dust particles up to 75 µm in size. Comparisons of road-deposited sediments at traffic curves or traffic light intersections revealed significantly higher concentrations of tire particles compared to areas such as parks [55]. For most plants, selenium concentrations in wet mass typically do not exceed about 0.1 mg·kg^−1^, except under specific conditions, for example, in areas with high selenium content in soil or in selenium hyperaccumulators [45].

The results of the Kruskal–Wallis test indicate differing patterns among the analyzed biological samples. In the case of urine samples, the highest number of statistically significant differences was observed between the settlement of Čepin and most of the other investigated locations (with the exception of Vladislavci), which may be attributable to somewhat higher median values. In contrast, for hair samples, the greatest number of statistically significant differences was recorded between Dalj and the remaining sites, primarily due to lower median values. For serum samples, it can be concluded that both the median selenium concentrations and the 75th percentiles in urban areas (Slavonski Brod, Vinkovci and Vukovar) were lower compared to those in the other investigated locations. Consequently, the largest number of statistically significant differences was found between these three cities and most of the locations in the vicinity of Osijek. Each biological sample (urine, serum, and hair) showed distinct patterns of differences, with no regularity in concentrations across locations.

The cluster analysis of selenium concentrations in hair samples demonstrated a clear spatial differentiation, with rural sites forming a distinct cluster from urban locations. This clustering pattern suggests that environmental or dietary factors influencing selenium exposure differ between rural and urban populations, possibly due to local food sources and different anthropogenic influences. Bio-fortification, defined as the process of enhancing selenium concentrations in crops and livestock through soil and plant management, is currently considered the most reliable and safe strategy for human selenium supplementation. As a result of both dietary intake and environmental exposure, it is therefore expected that selenium accumulates most prominently in hair samples. A comparable pattern was observed in urine samples, with urban and rural locations largely separated, although the clustering was not as pronounced as in hair. The less distinct separation observed in urine compared to hair may reflect differences in the kinetics of element accumulation and excretion. While hair represents a long-term record of exposure, incorporating intake over weeks or months, urine primarily reflects more recent or short-term exposure. Therefore, the spatial pattern is more pronounced in hair, whereas in urine it appears less clearly separated. The results for serum samples were very similar to those observed for urine, showing a comparable clustering pattern. While the clustering patterns observed in urine and serum were very similar, reflecting comparable short-term exposure profiles and acute exposure levels, the pattern in hair differed and likely reflects the fact that hair accumulates metals over a longer period.

PCA revealed the interrelationships among variables, namely, the concentrations in biological and environmental samples, as well as among the objects (sampling locations). This approach provided insights into how specific locations and sample types contribute to the overall variability, highlighting patterns not readily apparent from cluster analysis alone. PCA yielded results indicating a clearer separation of sampling locations into urban (Vinkovci, Vukovar, Slavonski Brod and Našice), mixed (Dalj and Osijek), and two distinctly rural sites (Vladislavci and Čepin) which were clearly distinguished from the others. These rural locations were characterized by higher metal concentrations in both biological samples and soil, highlighting their distinct environmental and exposure profiles. However, the highest selenium concentrations in plants were observed in urban locations, where plant samples were predominantly collected from city centers, highlighting the influence of urban environmental conditions on selenium accumulation. The elevated selenium concentrations observed in urban plants, particularly along roadsides and city centers, can be attributed primarily to atmospheric deposition from anthropogenic sources. Key contributors may include the combustion of coal and other fossil fuels, emissions from industrial facilities and the incineration of rubber, paper and municipal waste, all of which release selenium compounds that accumulate in urban plants.

## 5. Conclusions

To the best of our knowledge, this study represents the first comprehensive assessment of selenium concentrations in both biological and environmental samples in Croatia. The obtained results indicate that median selenium concentrations in the analyzed biological samples are comparable to those reported for populations residing in areas without significant anthropogenic selenium contamination, suggesting an overall adequate environmental selenium status. Although increased selenium concentrations were observed in dandelion samples collected from urban areas, these findings were not accompanied by elevated selenium concentrations in the corresponding soil samples, indicating that factors other than soil selenium content may influence selenium accumulation in plants. The integration of biological and environmental analyses provided a broader understanding of selenium distribution and potential exposure pathways within the studied population. These findings provide valuable baseline data for future monitoring of selenium status, environmental exposure assessment, and the development of public health and nutritional strategies in Croatia.

## Figures and Tables

**Table 1:** Descriptive statistics for urine samples.

Variable	Descriptive Statistics, Urine (µg·L^−1^)					
	Valid N	Median	Minimum	Maximum	Percentile 25.00	Percentile 75.00
Vladislavci	88	31.90	2.36	162.61	10.99	54.90
Dalj	106	15.71	2.20	130.54	9.19	36.90
Čepin	51	37.15	6.02	96.61	26.49	50.80
Našice	82	17.15	2.03	73.52	9.89	29.23
Osijek	64	25.64	2.64	111.85	15.23	34.98
Vukovar	51	15.02	2.19	46.88	10.62	20.10
Slavonski Brod	31	21.25	6.94	45.77	13.74	27.57
Vinkovci	23	19.21	3.15	43.09	15.36	28.38

**Table 2:** Descriptive statistics for serum samples.

Variable	Descriptive Statistics, Serum (µg·L^−1^)					
	Valid N	Median	Minimum	Maximum	Percentile 25.00	Percentile 75.00
Vladislavci	88	94.18	10.69	248.60	76.21	115.50
Dalj	106	104.10	16.56	358.40	77.52	126.20
Čepin	51	114.05	12.86	221.90	93.56	137.05
Našice	82	100.31	16.80	175.80	90.56	114.24
Osijek	64	111.60	13.54	264.80	83.21	141.68
Vukovar	51	87.35	64.37	118.03	79.96	92.42
Slavonski Brod	31	81.96	61.35	118.34	77.52	90.25
Vinkovci	23	80.76	66.10	114.82	76.72	87.17

**Table 3:** Descriptive statistics of hair samples.

Variable	Descriptive Statistics, Hair (µg·g^−1^)					
	Valid N	Median	Minimum	Maximum	Percentile 25.00	Percentile 75.00
Vladislavci	88	0.46	0.02	10.62	0.27	0.86
Dalj	106	0.21	0.06	1.04	0.15	0.32
Čepin	51	0.40	0.05	3.60	0.31	0.60
Našice	82	0.32	0.06	4.91	0.21	0.44
Osijek	64	0.35	0.11	1.98	0.21	0.48
Vukovar	51	0.28	0.05	0.62	0.20	0.37
Slavonski Brod	31	0.38	0.06	0.53	0.25	0.44
Vinkovci	23	0.33	0.01	0.48	0.23	0.40

**Table 4:** Descriptive statistics of soil samples.

Variable	Descriptive Statistics, Soil (mg·kg^−1^)					
	Valid N	Median	Minimum	Maximum	Percentile 25.00	Percentile 75.00
Vladislavci	6	0.42	0.29	0.58	0.32	0.53
Dalj	6	0.46	0.38	0.48	0.38	0.48
Čepin	6	0.51	0.27	0.65	0.27	0.65
Našice	6	0.38	0.28	0.40	0.28	0.40
Osijek	6	0.39	0.24	0.59	0.24	0.59
Vukovar	6	0.32	0.21	0.51	0.27	0.36
Slavonski Brod	6	0.33	0.16	0.60	0.27	0.38
Vinkovci	6	0.37	0.11	0.51	0.28	0.43

**Table 5:** Descriptive statistics of dandelion samples.

Variable	Descriptive Statistics, Dandelion (µg·kg^−1^)					
	Valid N	Median	Minimum	Maximum	Percentile 25.00	Percentile 75.00
Vladislavci	6	6.55	5.27	9.03	5.86	7.49
Dalj	6	7.58	7.17	37.37	7.32	22.53
Čepin	6	9.03	3.77	45.99	3.90	30.02
Našice	6	7.37	4.59	11.66	5.28	7.95
Osijek	6	6.86	6.08	7.78	6.18	7.61
Vukovar	6	41.58	13.83	672.39	29.92	76.91
Slavonski Brod	6	77.09	20.47	260.18	33.21	130.87
Vinkovci	6	84.41	14.88	648.12	32.82	185.13

## Data Availability

The data presented in this study are available on request from the corresponding author due to ethical and privacy restrictions, as the dataset contains information that could potentially compromise participant confidentiality.

## Supplementary Materials

The following supporting information can be downloaded at: https://www.mdpi.com/article/10.3390/jox16040123/s1, Table S1: URINE; Table S2: SERUM; Table S3: HAIR.
