# Supplementary material for: Environmental and Population Biomonitoring of Selenium in Eastern Croatia
Source: J Xenobiot. 2026 Jul 2;16(4):123. doi: 10.3390/jox16040123 (PMC13398239; doi:10.3390/jox16040123)
Supplement: Supplementary file 1 [file jox-16-00123-s001.zip › jox-4360186-supplementary.pdf]

Supplementary Materials: Environmental and Population Biomonitoring of Selenium in Eastern Croatia

Zvonimir Užarević, Martina Šrajer Gajdošik, Elvira Kovač-Andrić, Lidija Kalinić, Mihaela Vranješ Delać, Dinko Puntarić, Eda Puntarić, Domagoj Vidosavljević, Mario Begović and Vlatka Gvozdić

Table S1. URINE.

Depend.: urin	Multiple Comparisons p values (2-tailed); urin, Independent (grouping) variable: lokacije Kruskal-Wallis test: H (7, N= 459) =57.57965 p =.0000							
	1 R:260.47	2 R:202.17	3 R:327.39	4 R:168.14	5 R:249.45	6 R:172.96	7 R:230.74	8 R:218.09
1		0.064608	0.116133	0.004084	1.000000	0.004976	1.000000	1.000000
2	0.064608		0.000001	1.000000	0.682417	1.000000	1.000000	1.000000
3	0.116133	0.000001		0.000000	0.048843	0.000000	0.038561	0.028984
4	0.004084	1.000000	0.000000		0.045633	1.000000	1.000000	1.000000
5	1.000000	0.682417	0.048843	0.045633		0.059559	1.000000	1.000000
6	0.004976	1.000000	0.000000	1.000000	0.059559		1.000000	1.000000
7	1.000000	1.000000	0.038561	1.000000	1.000000	1.000000		1.000000
8	1.000000	1.000000	0.028984	1.000000	1.000000	1.000000	1.000000	

Table S2. SERUM.

Depend.: Serum	Multiple Comparisons p values (2-tailed); Serum, Independent (grouping) variable: Lokacije Kruskal-Wallis test: H (7, N= 496) =60.84235 p =.0000							
	1 R:217.19	2 R:254.85	3 R:322.70	4 R:276.40	5 R:306.84	6 R:190.67	7 R:167.55	8 R:150.00
1		1.000000	0.000807	0.199203	0.003931	1.000000	1.000000	1.000000
2	1.000000		0.153362	1.000000	0.614253	0.240564	0.079864	0.041184
3	0.000807	0.153362		1.000000	1.000000	0.000092	0.000056	0.000045
4	0.199203	1.000000	1.000000		1.000000	0.022280	0.008836	0.005200
5	0.003931	0.614253	1.000000	1.000000		0.000440	0.000250	0.000189
6	1.000000	0.240564	0.000092	0.022280	0.000440		1.000000	1.000000
7	1.000000	0.079864	0.000056	0.008836	0.000250	1.000000		1.000000
8	1.000000	0.041184	0.000045	0.005200	0.000189	1.000000	1.000000	

Table S3. HAIR.

Depend.: Hair	Multiple Comparisons p values (2-tailed); Hair, Independent (grouping) variable: Lokacija Kruskal-Wallis test: H (7, N= 496) =73.27181 p =.0000							
	1<br>R:321.47	2<br>R:163.34	3<br>R:303.96	4<br>R:254.94	5<br>R:260.47	6<br>R:209.04	7<br>R:271.81	8<br>R:238.65
1		0.000000	1.000000	0.069830	0.268309	0.000233	1.000000	0.381207
2	0.000000		0.000000	0.000389	0.000521	1.000000	0.005888	0.625842
3	1.000000	0.000000		1.000000	1.000000	0.023096	1.000000	1.000000
4	0.069830	0.000389	1.000000		1.000000	1.000000	1.000000	1.000000
5	0.268309	0.000521	1.000000	1.000000		1.000000	1.000000	1.000000
6	0.000233	1.000000	0.023096	1.000000	1.000000		1.000000	1.000000
7	1.000000	0.005888	1.000000	1.000000	1.000000	1.000000		1.000000
8	0.381207	0.625842	1.000000	1.000000	1.000000	1.000000	1.000000	
